# Natural Language Processing for Enhanced Clinical Decision Support in Allergy Verification for Medication Prescriptions

**DOI:** 10.1016/j.mcpdig.2025.100244

**Published:** 2025-06-10

**Authors:** Juan Pablo Botero-Aguirre, Michael Andrés García-Rivera

**Affiliations:** a Department of Artificial Intelligence, Hospital Pablo Tobón Uribe, Colombia; b Department of Pharmaceutical Services, Hospital Pablo Tobón Uribe, Colombia

## Abstract

**Objective:**

To develop and validate a named entity recognition (NER) model based on BERT-based model trained on Spanish-language corpor, for extracting allergy-related information from unstructured electronic health records.

**Patients and Methods:**

The model was fine-tuned using 16,176 manually annotated allergy-related entities from anonimized patient records (hospitalized patients between January 1, 2021, and June 30, 2024). The data set was divided into training (80%) and testing (20%) subsets, and model performance was evaluated using accuracy, recall, and F1 score. The validated model was applied to another data set with 80,917 medication prescriptions from 5859 hospitalized patients with at least one prescribed medication (during August and September 2024) to detect potential prescription errors. Sensitivity, specificity, and Cohen κ were calculated using manual expert review as the gold standard.

**Results:**

The model achieved an accuracy of 87.28% and an F1 score of 0.80. It effectively identified medication names (F1=0.91) and adverse reactions (F1=0.85) but struggled with recommendation-related entities (F1=0.29). The model detected prescription errors in 0.96% of cases, with a sensitivity of 75.73% and specificity of 99.98%. The weighted κ score (0.7797) indicated substantial agreement with expert annotations.

**Conclusion:**

The BERT-based model trained on Spanish-language corpora–based NER model demonstrated strong performance in identifying nonallergic cases (specificity, 99.98%; negative predictive value, 99.97%) and showed promise for clinical decision support. Despite moderate sensitivity (75.73%), these results highlight the feasibility of using Spanish-language NER models to enhance medication safety.

Medication prescription is a critical component of health care that requires precision to prevent avoidable adverse events. Computerized provider order entry systems are a feature of many Health Information Systems that have improved patient safety by enabling automated allergy verification through clinical decision support (CDS) systems. However, the effectiveness of these systems relies on the availability of structured and standardized data within the electronic health record (EHR)^1^ and the rate of adoption and acceptance of these systems by health care providers is low owing to financial, organizational, technological, and user-related factors.^2^^,^^3^

In practice, many allergy records are documented as free-text, limiting the ability of CDS systems to identify and alert physicians about potential conflicts between documented allergies and prescribed medications.^4^^,^^5^ This information gap introduces a significant risk to patient safety because allergies recorded in free-text do not consistently trigger automated alerts. Although medication errors related to allergies occur in a relatively small fraction of cases (0.25%), they can lead to severe adverse events with considerable impact on patient morbidity and health care costs and represent an important cause of preventable adverse drug events (ADEs) in the health care setting.^6^^,^^7^

Although the use of CDS and free-text has not been recommended owing to the technical difficulties,^8^ natural language processing (NLP) has emerged as a promising solution for extracting clinical information from unstructured text and enhancing its integration with decision support systems.^9^, ^10^, ^11^ In particular, deep learning–based models such as bidirectional encoder representations from transformers (BERT) and its variants have demonstrated superior performance in clinical text classification and medical entity recognition.^12^, ^13^, ^14^, ^15^, ^16^ The ability of BERT to understand context and semantics at a granular level makes it an ideal candidate for processing complex clinical narratives. This capability is critical for enhancing CDS for allergy verification, potentially reducing medication errors and improving patient safety.^17^, ^18^, ^19^

Transformer-based architectures, particularly BERT, have significantly advanced NLP by achieving state-of-the-art results in tasks such as named entity recognition (NER), text classification, and question answering.^20^ The ability of BERT to model bidirectional contextual dependencies makes it especially well-suited to understanding the complex linguistic structures found in clinical texts.^21^

Although NLP has advanced considerably in English-language clinical texts, less-resourced languages—such as Spanish, Italian, German, and Korean—require language-adapted models and domain-specific annotated corpora. Originally trained on English data, BERT underperforms in other languages unless further adapted.^12^ BERT-based model trained on Spanish-language corpora (BETO), a Spanish-language BERT variant, improves upon multilingual models in general NLP tasks,^22^ but its clinical applications remain limited. Effective NER in these settings often relies on fine-tuning with annotated health data, as shown in Italian,^23^ German,^24^ and Korean^25^ versions. Despite its global use, Spanish versión lacks high-quality clinical corpora; most work has focused on structured data rather than on unstructured narratives,^26^ leaving key safety-relevant information—such as allergies—underexploited.

This study aimed to addresses the gap in Spanish-language clinical NLP by developing and validating a NER model based on BETO for extracting allergy-related information from unstructured EHRs. The extracted information is then used to detect potential conflicts between recorded allergies and prescribed medications, thus contributing to medication safety in hospitalized patients. To our knowledge, this is the first implementation of a Spanish-language model for this task that has been evaluated both on an annotated corpus and in a real-world hospital setting to detect medication-allergy conflicts and support clinical decision making.

## Patients and Methods

### Data Sources and Study Sample

This study was performed with the approval of our institutional review board (IRB protocol number 2024.047, meeting minute 8/2024) and following Colombian legislation about data in human research. A waiver of informed consent was granted by the IRB because the study involved retrospective analysis of fully anonymized clinical data and posed minimal risk to participants. For this study, an anonymized data set was constructed, consisting of allergy histories to medications from hospitalized patients between January 2021 and June 2024, resulting in a corpus of 16,176 entities. To identify and classify the relevant entities, NER was used. This process required the definition of specific sequence labels for each word in the text. These labels enable the classification of words and the preparation of the data set for model training.

The labels defined for this study were as follows:

1. B-Medicamento and I-Medicamento: identify the name of the medication.
2. B-RAM and I-RAM: indicate adverse drug reactions.
3. B-NoRAM and I-NoRAM: highlight information unrelated to allergic reactions to the medication.
4. B-Recomendaciones and I-Recomendaciones: identify recommendations related to the administration of medications.
5. O: assigned to irrelevant words in the context of allergy histories, such as dates or text unrelated to allergies.

On the basis of the data set and defined labels, a manual annotation process was conducted manually by a clinical pharmacist trained in medical informatics. Using a custom-developed guideline aligned with CONLL-2003 standards, the annotator applied BIO tags to allergy-related entities, including medications, reactions, nonrelevant mentions, and recommendations. Annotated entities were reviewed through an internal quality check process focused on consistency and medical plausibility. In this format, each line contains a word, a space, and its corresponding label in the BIO scheme: B (beginning of an entity), I (inside a multitoken entity), and O (outside relevant entities). This scheme ensures consistency and facilitates model training.

### Model Fine-Tuning Strategy and Mitigation of Catastrophic Forgetting

The approach adopted for NER in patient allergy histories involved fine-tuning the last layer of the BETO model’s neural network. BETO is a BERT-based model trained on a Spanish-language corpus and built on the transformer architecture. BETO has a size comparable with BERT-Base and was pretrained using the whole-word masking technique.

To adapt the BETO model to the domain of clinical allergy narratives, the study applied a supervised fine-tuning process using the annotated data set. Acknowledging the risk of catastrophic forgetting (a phenomenon in which pretrained models lose previously acquired general knowledge when exposed to a specialized task), a conservative fine-tuning strategy was adopted to preserve the model’s general language understanding while enhancing its performance on the specific NER task. The current model does not include a component for assertion status detection (eg, negation or speculation). All extracted entities are assumed to be affirmed mentions.

A low learning rate of 2 × 10^−5^ and a batch size of 16 were used to ensure gradual parameter updates. Training was limited to 5 epochs to minimize overfitting to the relatively small clinical corpus, and a weight decay of 0.01 was applied to prevent excessive parameter shifts. Fine-tuning was conducted using a stratified 80/20 split of the annotated corpus, ensuring balanced representation of all entity types across training and evaluation subsets.

Only the final classification layer of the model was significantly updated, whereas the core transformer layers were largely preserved. This selective adaptation strategy, commonly referred to as partial fine-tuning, was intended to maintain the semantic richness of the original language model. Training dynamics, including loss curves and label-wise performance across epochs, were monitored, and no evidence of catastrophic forgetting was observed.

The training was conducted on the Google Colab platform using a TPU as the training hardware. The implementation of this process relied on the following libraries and their respective versions: transformers (versión 4.41.2) for model handling, data sets (versión 2.20.0) for data management, TensorFlow (versión 2.15.0) as the core deep learning framework, accelerate (versión 0.31.0) to optimize training, along with numpy (versión 1.26.4), pandas (versión 2.2.2), and matplotlib (versión 3.8.3) for data manipulation and visualization. The parameters used for fine-tuning included a batch size of 16, a learning rate of 2 × 10^−5^, 5 training epochs, and a weight decay of 0.01. The annotated corpus was divided into 2 subsets: training (80%) and testing (20%), ensuring that the distribution of labels was preserved across each subset.

### Clinical Validation

A separate data set comprising 80,917 medication prescriptions from 5859 hospitalized patients (all hospitalized patients who had at least 1 prescribed medication during August and September 2024) was used to validate the clinical impact of the NER model in detecting allergy-related prescription errors. Each drug was matched to the documented allergy record for conflict detection. This data set was independent from the annotated corpus used for training. We applied the NER model to extract allergy-related entities from patient records to crossreferencing these extracted entities with prescribed medications to identify potential prescription errors where a patient was prescribed a medication to which they were labeled as allergic. The model’s results were compared against a manual review conducted by a clinical pharmacist, who served as the gold standard.

### Statistical Analyses

The performance of the NER model was evaluated using standard metrics, including accuracy, recall, and F1 score (métrica de evaluación del modelo), computed at the entity level using the seqeval.metrics (versión 1.2.2) and sklearn.metrics (versión 1.4.0) Python packages. Metrics were reported globally and by entity label (eg, medication and adverse reaction), enabling detailed analysis of the model’s ability to identify clinically relevant concepts compared with manually annotated data by clinical pharmacists. To assess real-world clinical utility, a validation phase was conducted in which the model’s outputs were applied to detect medication-allergy conflicts in hospitalized patients. These results were evaluated using sensitivity, specificity, and the Cohen κ adjusted for class imbalance,^27^ based on comparison with expert-reviewed outcomes. All metrics were calculated with 95% CIs.

A direct comparison with existing baseline models was not feasible because there are currently no publicly available NER systems specifically trained to extract allergy-related entities from Spanish clinical narratives. Although traditional baselines such as rule-based or Conditional Random Fields models were considered, the lack of standardized terminologies, annotated corpora, and evaluation benchmarks in Spanish limited the interpretability and generalizability of such comparisons. This constraint is consistent with previous reports highlighting the scarcity of domain-specific NLP resources for Spanish and other less-resourced languages in clinical contexts.^28^^,^^29^ Given these limitations, the study prioritized dual evaluation: intrinsic performance based on annotated data and extrinsic validation in a real-world hospital setting.

A visual summary of the study workflow—including model training, intrinsic evaluation, and external clinical validation—is presented in Figure. The diagram illustrates the data flow from the annotated corpus through BETO-based fine-tuning to the final application in real-world allergy–medication conflict detection.

**Figure:**
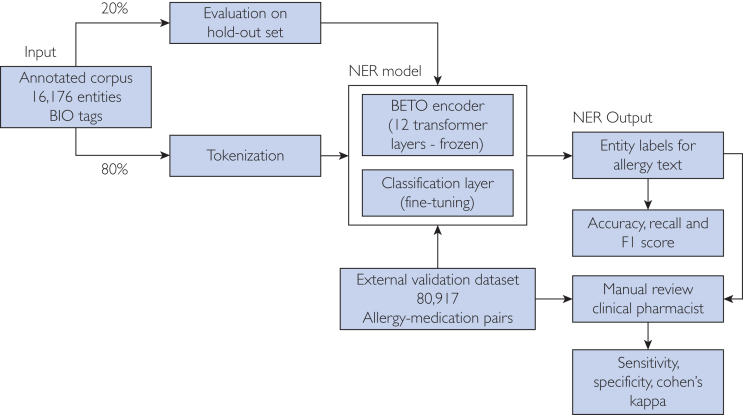
Study design for BETO-based named entity recognition (NER) model development and clinical validation.

## Results

The intrinsic evaluation of the NER model was evaluated using the 20% of the corpus (n=3235 entities). The overall accuracy was 87.28% (95% CI, 86.88%-87.68%), with specificity of 82.25% (95% CI, 81.59%-82.91%) and F1 score of 0.80 (95% CI, 0.80-0.81). The performance by entity shows 4 of the 9 categories achieved F1 scores above 0.77 based on token-level BIO-tag classification across entity types: medications, adverse reactions, unrelated information (NoRAM), and recommendations (Table).

**Table:** Performance of the BETO-Based NER Model in Extracting Allergy-Related Entities From Annotated Clinical Texts

Performance metric	Accuracy (%) (95% CI)	Recall (%) (95% CI)	F1 score (95% CI)
Overall	87.28 (86.88-87.68)	82.25 (81.59-82.91)	0.80 (80.28-81.51)
B-Medicamento	91.80 (89.10-94.60)	91.70 (90.30-93.00)	0.91 (0.90-0.93)
I-Medicamento	78.90 (73.00-84.80)	57.40 (52.50-62.30)	0.66 (0.62-0.70)
B-RAM	85.50 (83.50-87.40)	85.40 (84.70-86.00)	0.85 (0.84-0.86)
I-RAM	68.70 (62.80-74.60)	56.50 (53.10-59.80)	0.61 (0.59-0.64)
B-NoRAM	84.30 (79.40-89.20)	77.50 (75.20-79.80)	0.80 (0.79-0.80)
I-NoRAM	74.10 (70.00-78.20)	72.40 (68.80-76.10)	0.73 (0.72-0.74)
B-Recomendaciones	60.00 (32.20-87.8)	20.00 (20.00-20.00)	0.29 (0.26-0.32)
I-Recomendaciones	0.00 (0.00-0.00)	0.00 (0.00-0.00)	0.00 (0.00-0.00)
O	89.00 (88.40-89.60)	92.70 (82.40-93.00)	0.908 (0.90-0.91)

Metrics are reported per entity type based on BIO tagging. These results pertain to the NER development phase and do not reflect downstream error detection metrics.

The labels B-Medicamento and I-Medicamento are used to identify the name of the medication. B-RAM and I-RAM indicate adverse drug reactions, while B-NoRAM and I-NoRAM highlight information unrelated to allergic reactions to the medication. The labels B-Recomendaciones and I-Recomendaciones are used to identify recommendations related to the administration of medications. The label O is assigned to irrelevant words in the context of allergy histories, such as dates or text unrelated to allergies.

NER, named entity recognition.

### Validation

A total of 80,917 medication prescriptions from 5859 hospitalized patients were analyzed. Prescription errors were detected in n=776.8 of the evaluated prescriptions. The analysis yielded 78 true positives, 80,795 true negatives, 19 false positives, and 25 false negatives. Sensibility and specificity were 75.73% (95% CI, 66.62%-82.98%) and 99.98% (95% CI, 99.96%-99.98%), respectively, for the detection of the prescription of a medication to a patient who was labeled as allergic. The negative predictive value was 99.97% (95% CI, 99.95%-99.98%), with a positive predictive value of 80.41% (95% CI, 71.42%-87.09%). Overall, 44 of 80,917 results (0.05%; 95% CI, 0.04%-0.07%) were discrepant compared with manual review by a human. The model achieved a weighted κ of 0.7797 (95% CI, 0.7754-0.7840).

The analysis of false negatives revealed 25 cases where the model failed to correctly identify an allergy-related entity. The most frequently missed entities included ondansetron (4 cases), spironolactone (3 cases), and piperacillin/tazobactam (2 cases). The analysis of false positives identified 19 cases where the model incorrectly classified nonallergic terms as allergens. The most frequently misclassified entities included hydromorphone (12 cases), acetaminophen (3 cases), and dypirone–metamizole (1 case).

## Discussion

Our results indicate that the trained NER model effectively classifies named entities in patients’ medication-allergy records. Specifically, the model demonstrated strong performance in identifying medication-related entities (accuracy of 94.64% for B-Medication label) and adverse drug reactions (accuracy of 86.62% for B-RAM label). However, its performance was significantly lower for recommendation-related entities, with an accuracy of only 50.00% for B-Recommendaciones and 0.00% for I-Recommendaciones. This discrepancy suggests a potential imbalance in the training data, likely owing to the low frequency of these labels in the data set. Additionally, it may reflect an inherent difficulty in distinguishing recommendation-type entities within clinical text, highlighting the need for data set augmentation or model refinement to enhance classification in underrepresented categories.^25^

These findings align with previous research on NER models in clinical settings, which have reported similar trade-offs between precision and recall due to challenges associated with unstructured medical text.^30^^,^^31^ Studies have demonstrated that deep learning–based transformer models, such as BERT and its variants, outperform rule-based and dictionary-based approaches in recognizing complex medical entities, particularly across diverse linguistic contexts.^15^ However, class imbalance, ambiguous entity boundaries, and domain-specific terminology frequently contribute to reduced sensitivity, as observed in our study.^32^

When analyzing performance by entity type, 4 of 9 categories achieved F1 scores above 77%, indicating that the model was generally effective across most classifications. However, the lower sensitivity suggests that certain allergic reactions—particularly those with ambiguous phrasing or rare terminology—were more challenging for the model to identify. Similar findings have been reported in previous NLP studies focused on medication safety and adverse drug reaction detection, where models struggle with nonstandardized documentation styles.^33^^,^^34^ The weighted κ of 0.7797 indicates substantial agreement between the model and expert annotations, reinforcing the model’s consistent performance across different entity classifications.

The low number of true positives in our validation reflects the real-world rarity of confirmed drug allergies in hospitalized patients. Although EHRs often report allergy rates ranging from 10% to 35%, many entries are unverified or refer to nonimmunologic reactions.^35^ For instance, a study across 2 tertiary hospitals in Boston found that 35.5% of inpatients had at least 1 recorded drug allergy—most commonly to penicillins (12.8%) and sulfonamides (7.4%)—yet many were later deemed inaccurate or outdated.^36^ In contrast, studies using allergy testing report that up to 90% of penicillin allergy labels are incorrect.^37^ In our data set, 78 true positives were identified of 80,917 prescriptions, which aligns with the lower end of validated allergy prevalence. Although infrequent, these events pose serious risks. The model’s ability to detect them with high specificity supports its integration into CDS systems focused on high-impact, preventable errors.

Automated extraction of allergy-related entities from unstructured clinical text enables real-time detection of potential medication-allergy conflicts. This capability could be integrated into CDS systems to alert health care providers, which is particularly relevant given that medication errors related to allergies remain a leading cause of preventable ADEs.^6^^,^^7^ A major advantage of our system is its ability to process free-text allergy documentation, which remains prevalent in EHRs.

Overall, our findings highlight the potential of clinical NLP in improving patient safety, enhancing allergy documentation, and reducing preventable ADEs. Future work should focus on addressing the identified limitations by improving model sensitivity, mitigating class imbalance, and refining entity classification for underrepresented categories. Additionally, integrating structured data sources and optimizing postprocessing strategies could further enhance performance. By addressing these challenges, this research lays the foundation for scalable, real-world implementations of AI-driven allergy detection systems in health care.

### Limitations

Despite the model’s strong overall performance, several limitations should be considered. First, its moderate sensitivity (75.73%) may reflect the inherent ambiguity and variability in clinical documentation, where allergens and reactions are often recorded using nonstandardized terms, abbreviations, or misspellings. This inconsistency can hinder the model’s ability to reliably recognize entities, particularly when allergic reactions are implicit or context dependent, requiring semantic interpretation beyond standard NER.^30^^,^^31^

Second, the model relies exclusively on free-text allergy documentation, which varies in quality and completeness across EHRs. Unlike structured coding systems (eg, SNOMED CT or RxNorm), free-text entries often include fragmented or negated expressions that increase the risk of misclassification.^8^^,^^26^ Integrating structured data sources or incorporating rule-based postprocessing could help mitigate these challenges.^4^

Third, although the model achieved high specificity (99.98%), the 19 false positives observed may lead to unnecessary alerts when deployed in a CDS system. This could contribute to alert fatigue, potentially undermining clinician trust.^38^ To address this, future work should explore threshold calibration or multistage filtering strategies to prioritize high-confidence, clinically relevant alerts.

Fourth, the annotation process did not include interannotator agreement assessment, as labeling was conducted by a single domain expert. Although this ensured clinical validity, the lack of formal agreement metrics (eg, Cohen κ) limits our ability to evaluate annotation consistency and data set reliability. In future iterations, multiple annotators and quantitative agreement measures will be considered.

Fifth, neither the annotated data set nor the fine-tuned model can currently be made publicly available owing to institutional data-use restrictions and residual reidentification risks inherent to free-text clinical narratives, particularly in underresourced languages like Spanish. Moreover, the lack of publicly available annotated corpora and data-sharing constraints prevented external validation, limiting the generalizability of the model to other institutions.

Finally, the model does not currently support assertion detection; it cannot distinguish between affirmed, negated, or uncertain allergy mentions. This limits its direct applicability in clinical decision making, where understanding the certainty of statements is crucial. Incorporating assertion classification is a planned enhancement for future model versions.

## Conclusion

This study demonstrates the feasibility and clinical relevance of using a NER model based on BETO to extract allergy-related entities from unstructured clinical text and crossreference them with prescribed medications to identify potential medication-allergy conflicts. Despite operating in a low-resource language context, the model achieved excellent performance in key areas, including high specificity (99.98%) and negative predictive value (99.97%), highlighting its strong reliability in ruling out nonallergic cases and minimizing unnecessary alerts. Although the model’s moderate sensitivity (75.73%) indicates room for improvement in capturing all true allergic reactions, these results confirm the potential of Spanish-language transformer models as a foundational tool for enhancing medication safety through CDS.

## Potential Competing Interests

The authors declare that Hospital Pablo Tobón Uribe provided funding for the conduct of this study. However, the hospital had no role in the study design, data collection and analysis, manuscript preparation, or the decision to publish the results.

## Ethics Statement

This study was performed with the approval of our institutional review board (IRB protocol number 2024.047, meeting minute 8/2024) and following Colombian legislation about data in human research. A waiver of informed consent was granted by the IRB because the study involved retrospective analysis of fully anonymized clinical data and posed minimal risk to participants.

## Author Contributions

Juan Pablo Botero Aguirre: conceptualisation, data curation, formal analysis, investigation, methodology, writing – original draft, and writing – review & editing, supervisión.

Michael Andrés García Rivera: data curation, formal analysis, software, validation, visualisation, and writing – review & editing.
